# Three decades of cochlear implant failure and multiple reimplantation: a retrospective cohort study

**DOI:** 10.3389/fsurg.2026.1941723

**Published:** 2026-10-20

**Authors:** Luluh Alsughayer, Nezar Hamed, Thamer Alzahrani, Abdullah Alsarar, Rahma Sweedy, Turky AlHarbi, Wafaa S. Taishan, Afrah Alshalan, Farid Alzhrani

**Affiliations:** 1 Department of Otolaryngology-Head and Neck Surgery, College of Medicine, King Saud University, Riyadh, Saudi Arabia; 2 King Abdullah Ear Specialist Center (KAESC), King Saud University Medical City, King Saud University, Riyadh, Saudi Arabia; 3 Department of Otolaryngology-Head and Neck Surgery, King Faisal Specialist Hospital and Research Centre, Riyadh, Saudi Arabia; 4 College of Medicine, Imam Mohammed Ibn Saud Islamic University, Riyadh, Saudi Arabia; 5 Egyptian Ministry of Health (MOH), Cairo, Egypt; 6 Audiology-Speech Language Pathology, Sultan Bin Abdulaziz Humanitarian City, Riyadh, Saudi Arabia; 7 Department of Otolaryngology, Makkah Health Cluster, Makkah, Saudi Arabia; 8 Department of Otolaryngology, Head & Neck Surgery, College of Medicine, Jouf University, Skaka, Aljouf, Saudi Arabia

**Keywords:** auditory perception, cochlear implantation, electrode, green cochlea, prosthesis failure, reoperation, speech perception, sustainability

## Abstract

**Introduction:**

Cochlear implantation (CI) is a highly reliable intervention for sensorineural hearing loss; however, device failure may occur, limiting patient benefit unless revision surgery is performed. This study evaluated the long-term institutional prevalence and causes of CI failure, and assessed complications, audiological and speech outcomes, and their associated factors following multiple revision surgeries.

**Materials and methods:**

A retrospective cohort study identified 91 failed CI cases among 3,777 CIs performed at a single tertiary referral center between January 1993 and November 2024. Audiological and speech outcomes, including Pure Tone Average (PTA), Speech Reception Threshold (SRT), Speech Discrimination Score (SDS), Categories of Auditory Performance (CAP), and Speech Intelligibility Rating (SIR), were analyzed using Mixed Models for Repeated Measures (MMRM) with restricted maximum likelihood (REML) estimation.

**Results:**

The overall prevalence of CI failure was 2.41%, occurring predominantly in pediatric patients (96.7%). Hard device failure was the most common cause (68.8%), followed by medical (15.0%), soft (13.8%), and surgical (2.5%) failures. Revision surgery was performed once in 80 cases, twice in 8 patients, and three times in 2 cases. Reimplantation successfully preserved audiological performance across successive revisions, while CAP and SIR scores demonstrated significant additional improvement after the first revision compared with the initial implantation (*p* = 0.037 and *p* < 0.001, respectively). Reimplantation using the same electrode model was associated with better SDS after the first revision (*p* = 0.035), and better SRT (*p* = 0.022) and SIR (*p* = 0.001) after the second revision. An interval of >3 years between surgeries was associated with significantly higher post-revision SIR scores after both the first (*p* = 0.040) and second (*p* = 0.004) revisions.

**Conclusion:**

CI failure had a prevalence of 2.41% over more than three decades. Reimplantation is a salvage procedure that successfully restores auditory function after device failure, allowing for continuous language development. Electrode selection and revision strategy should remain tailored to each case.

## Introduction

Cochlear implantation (CI) has become the established treatment for patients with severe-to-profound sensorineural hearing loss, providing substantial improvements in auditory perception, speech understanding, and quality of life. Advances in implant technology, surgical techniques, and postoperative rehabilitation have resulted in excellent long-term outcomes with a high degree of device reliability. Nevertheless, complications related to the implant or the surgical procedure remain inevitable in a small proportion of recipients, occasionally resulting in device failure and the need for revision surgery or reimplantation to restore auditory function and maintain hearing rehabilitation (1, 2).

Revision CI is an uncommon procedure, with reported rates of approximately 4% of all implanted cases (3). Reported failure rates vary across studies and are influenced by several factors, including patient age, duration of implantation, and implant manufacturer (1).

The indications for revision surgery can be broadly categorized as device-related (hard or soft failure) and non-device-related (medical or surgical causes) (4). Across large clinical series, device failure remains the predominant indication for reimplantation, accounting for nearly three-quarters of revision procedures (1–3). Pediatric recipients are disproportionately affected, likely reflecting higher exposure to head trauma (5).

Overall, reimplantation can maintain or improve hearing and speech performance following revision surgery (6). Successful outcomes rely on meticulous surgical planning and execution, including careful management of intracochlear fibrosis or ossification, preservation of cochlear structures, appropriate electrode selection, and achievement of optimal electrode insertion while minimizing additional intracochlear trauma (7).

Although outcomes after a single revision procedure are well documented, evidence regarding patients who undergo multiple revision cochlear implantations remains limited. Recurrent device failure presents unique clinical challenges, as it is uncertain whether repeated reimplantation continues to preserve or improve audiological and speech outcomes or whether successive surgeries increase the risk of complications, technical difficulty, and healthcare costs. Furthermore, factors influencing outcomes post repeated device failure have not been comprehensively characterized.

Re-implantation is not a straightforward decision. Upon re-implantation, structural fibrotic changes within the cochlea might necessitate a change in electrode design, often switching to shorter and stiffer arrays, which can compromise optimal tonotopic neural stimulation and speech perception (8). Hence, should the patient be re-implanted with a similar type of electrode to his index procedure, or would a change of electrode provide superior results. Furthermore, resistance upon electrode re-insertion cannot guarantee the previously achieved insertion depth, and incomplete coverage can theoretically disrupt the efficiency of acoustic coding. We also hypothesise that earlier re-implantation may provide better outcomes than delayed re-implantation. Finally, the growing advances of technology of CI may influence the temporal changes in revision outcomes.

The present study evaluates the long-term prevalence, causes, and outcomes of cochlear implant failure over more than three decades at a high-volume tertiary referral center. In addition to assessing audiological and speech performance following repeated revision surgeries, we investigate factors associated with postoperative outcomes, including electrode selection, device upgrades, surgical timing, and multiple reimplantations.

## Methodology

### Study design and patient population

A retrospective cohort study was conducted at King Abdullah Ear Specialist Centre, Riyadh, Saudi Arabia. Medical records of all patients who experienced CI failure and subsequently underwent revision surgery between January 1993 and November 2024 were reviewed. Patients of all ages were eligible for inclusion. A total of 91 patients who underwent explantation with or without reimplantation during the study period were included in the analysis.

The study was conducted in accordance with the principles of the Declaration of Helsinki and was approved by the King Saud University Institutional Review Board (Approval No. E-23-8334). Owing to the retrospective nature of the study and the use of anonymized data, the requirement for informed consent was waived.

### Definitions

In the present cohort, revision surgery can refer to explantation and reimplantation of the CI. However, re-implantation was defined strictly as replacement of the device and electrode array within the same previously implanted ear. On this definition, 80 cases underwent CI explantation and re-implantation, and 11 cases underwent explantation of CI without re-implantation. Occasionally, primary implantation of the contralateral ear during the same surgical setting was performed (*n* = 29).

Hard failure was defined as confirmed malfunction of the internal cochlear implant device, verified by abnormal integrity testing and/or manufacturer analysis. Soft failure was defined as unexplained deterioration in auditory performance or abnormal auditory symptoms despite normal device integrity testing, after exclusion of medical, surgical, programming, and other identifiable causes. Revision procedures performed for medical or surgical indications—including infection, device extrusion, cholesteatoma, magnet-related complications, or electrode malposition—were classified separately as non-device-related causes of revision surgery (4).

### Data collection

Demographic, clinical, surgical, and audiological data were retrospectively extracted from the institutional CI database and electronic medical records at our tertiary CI center in 2024, including all cases between January 1993 and November 2024. Variables collected included age at implantation, gender, comorbidities, laterality of hearing loss and CI (including single-sided deafness), onset of hearing loss, implanted device model, electrode array type, indication for revision surgery, number of revision procedures, interval between surgeries, intraoperative findings (including incomplete electrode insertion and insertion resistance), and postoperative complications.

Audiological outcomes were assessed using Pure Tone Average (PTA), Speech Reception Threshold (SRT), and Speech Discrimination Score (SDS). PTA was used to assess the average hearing threshold (dB HL) across speech frequencies (500, 1,000, and 2,000 Hz). Based on patients age, we used Visual Reinforcement Audiometry (6 months–2 years), Conditioned Play Audiometry (2–5 years), or Conventional Audiometry (5 + years). SRT measured the lowest intensity (dB HL) where the patient can correctly identify 50% of the Arabic Spondaic words. SDS expresses the percentage (%) of phonetically balanced monosyllabic Arabic words correctly understood at a comfortable listening level (SRT + 30–40 dB). Monoaural assessment was ensured by plugging, muffling, and masking the non-test ear. For patients with a contralateral CI, the contralateral CI is turned off during audiological and speech assessment. However, for many non-verbal infant and children, SRT/SDS could not be done.

Functional hearing and speech outcomes were evaluated using the Categories of Auditory Performance (CAP-II) and Speech Intelligibility Rating (SIR). CAP measures the ability to detect, discriminate, and understand spoken sound in real-life situations. CAP-II is rated 0–9, where category 0 represents lack of awareness of environmental sounds, and category 9 represents the use of phone with unknown speaker in unpredictable context (9). Meanwhile, SIR measures speech production clarity, where the child's primary mode of everyday communication may be manual in category 1, while the child's connected speech is intelligible to all listeners in category 5 (10). Audiological and speech measures have been assessed frequently post-implantation. The score chosen for analyses was the latest score before re-implantation, which averaged 18 months post operatively.

### Statistical analysis

Descriptive statistics were performed for qualitative data using absolute and relative frequencies, while for quantitative outcome variables, the raw means and the model-based estimated marginal means (EMM) with the corresponding lower and upper limits of the 95% confidence interval were used. Cumulative incidence (prevalence) of the investigated events of interest was calculated with the corresponding Clopper–Pearson (exact) confidence interval recommended for the small sample sizes.

A mixed model for repeated measures (MMRM) was fitted to analyse the longitudinal change in each outcome measure over revision surgeries, adjusted for potential surgical characteristics as an interaction term. Followed by *post-hoc* Tukey-adjusted pairwise comparisons, accounting for multiple levels across the covariate addressed through each model. One fitted model was applied to adjust for each investigated surgical characteristic, including changing electrode type, depth of insertion, duration between surgeries, and upgrading status of the implant model. The multiple surgeries variable was treated as a categorical fixed-effect covariate by interaction with one of each of the mentioned investigated predictors. Multivariate modelling was avoided in this study because of the small sample size limitation.

This MMRM using the restricted maximum likelihood (REML) model was selected based on the unbalanced nature of the data, as not all cases had undergone all revision surgeries, in addition to incomplete records for some audiological and speech assessments. The compound symmetry covariance structure was assumed that requires only 2 variance parameters, supporting the robustness of the results in cases of small group sizes rather than the unstructured covariance, which is more likely to fail to converge in such cases. No random intercept or random slope was specified in MMRM, as the model can handle the within-subject error through the direct correlations between multiple observations within each subject. *P* values less than 0.05 were considered statistically significant, while the 95% confidence intervals were reported for interval estimates. All analyses were performed in R Statistical and Computing Platform (version 4.5.2).

## Results

Among 3,777 CI procedures performed at our tertiary center between January 1993 and November 2024, 91 cases experienced cochlear implant failure, yielding an overall failure prevalence of 2.41% (95% confidence interval: 1.94%–2.95%). Revision surgery with reimplantation was performed in 80 of the 91 patients, whereas the remaining 11 cases underwent explantation without reimplantation. Among the reimplanted patients, 8 (10.0%) required a second reimplantation because of recurrent device failure, and 2 (2.5%) subsequently underwent a third reimplantation. Relative to the entire CI cohort, the prevalence of patients requiring a second and third reimplantation was 0.21% (8/3,777) and 0.05% (2/3,777), respectively.

Among patients with CI failure, 46.15% had bilateral CIs but experienced failure in only one device (95% confidence interval: 35.64%–56.92%), whereas 40.66% had a unilateral implant with failure of the implanted device (95% confidence interval: 30.48%–51.47%). Bilateral device failure following bilateral implantation occurred in 13.19% of patients (95% confidence interval: 7.0%–21.9%).

### Demographic, clinical, and surgical characteristics

Table 1 summarizes the demographic, clinical, and surgical characteristics of the 91 patients who experienced CI failure following primary implantation. The cohort was predominantly pediatric (96.7%, *n* = 87), with a mean age at implantation of 4.3 ± 3.0 years. Only 3 adults underwent a single revision surgery, with a mean age at implantation of 45.8 ± 7.4 years. Gender distribution was relatively balanced, comprising 52 males (57.1%) and 39 females (42.9%). Hard device failure was the most common indication for revision surgery (68.8%), followed by medical causes (15.0%), soft device failure (13.8%), and surgical causes (2.5%).

**Table 1:** Baseline demographic, clinical, and surgical characteristics.

Patients characteristics	Failure post first CI	First reimplantation	Second reimplantation	Third reimplantation
	*N* = 91	*N* = 80	*N* = 8	*N* = 2
Demographics				
Pediatrics	87.0 (96.7%)	76.0 (96.2%)	8.0 (100.0%)	2.0 (100.0%)
Male	52.0 (57.1%)	45.0 (56.3%)	4.0 (50.0%)	2.0 (100.0%)
Side of hearing loss				
Bilateral	86.0 (94.5%)	76.0 (95.0%)	7.0 (87.5%)	2.0 (100.0%)
Left-sided	2.0 (2.2%)	2.0 (2.5%)	0.0 (0.0%)	0.0 (0.0%)
Right-sided	3.0 (3.3%)	2.0 (2.5%)	1.0 (12.5%)	0.0 (0.0%)
Side of CI				
Bilateral	54.0 (59.3%)	48.0 (60.0%)	5.0 (62.5%)	2.0 (100.0%)
Left-sided	21.0 (23.1%)	21.0 (26.3%)	2.0 (25.0%)	0.0 (0.0%)
Right-sided	16.0 (17.6%)	11.0 (13.8%)	1.0 (12.5%)	0.0 (0.0%)
Side of CI failure				
Bilateral	12.0 (13.2%)	11.0 (13.8%)	1.0 (12.5%)	0.0 (0.0%)
Left-sided	43.0 (47.3%)	42.0 (52.5%)	4.0 (50.0%)	2.0 (100.0%)
Right-sided	36.0 (39.6%)	27.0 (33.8%)	3.0 (37.5%)	0.0 (0.0%)
Cause of CI failure				
Hard	55.0 (68.8%)	54.0 (78.3%)	7.0 (100.0%)	2.0 (100.0%)
Soft	11.0 (13.8%)	10.0 (14.5%)	0.0 (0.0%)	0.0 (0.0%)
Medical	12.0 (15.0%)	3.0 (4.3%)	0.0 (0.0%)	0.0 (0.0%)
Surgical	2.0 (2.5%)	2.0 (2.9%)	0.0 (0.0%)	0.0 (0.0%)
Unspecified	11	11	1	0
Comorbidities and complications^a^				
Comorbidities	29.0 (31.9%)	20.0 (25.0%)	2.0 (25.0%)	1.0 (50.0%)
Complications	18.0 (19.8%)	12.0 (15.0%)	1.0 (12.5%)	1.0 (50.0%)
Electrode type				
Advanced bionics				
MS	6.0 (6.9%)	4.0 (5.2%)	0.0 (0.0%)	0.0 (0.0%)
SlimJ	4.0 (4.6%)	4.0 (5.2%)	0.0 (0.0%)	0.0 (0.0%)
MED-EL				
Flex24	7.0 (8.0%)	3.0 (3.9%)	0.0 (0.0%)	0.0 (0.0%)
Flex26	0.0 (0.0%)	1.0 (1.3%)	0.0 (0.0%)	0.0 (0.0%)
Flex28	37.0 (42.5%)	26.0 (33.8%)	2.0 (25.0%)	0.0 (0.0%)
Form24	12.0 (13.8%)	22.0 (28.6%)	5.0 (62.5%)	1.0 (50.0%)
Medium	6.0 (6.9%)	2.0 (2.6%)	0.0 (0.0%)	0.0 (0.0%)
Standard	7.0 (8.0%)	4.0 (5.2%)	0.0 (0.0%)	0.0 (0.0%)
Cochlear				
CA	7.0 (8.0%)	6.0 (7.8%)	0.0 (0.0%)	0.0 (0.0%)
SS	1.0 (1.1%)	5.0 (6.5%)	1.0 (12.5%)	1.0 (50.0%)
Missing data	4	3	0	0
Changing electrode type in revision surgeries				
Changed	Not applicable	32.0 (43.2%)	4.0 (50.0%)	1.0 (50.0%)
Not changed	Not applicable	42.0 (56.8%)	4.0 (50.0%)	1.0 (50.0%)
Missing data	Not applicable	6	0	0
Surgical approach				
Cochleostomy	9.0 (9.9%)	11.0 (13.8%)	0.0 (0.0%)	1.0 (50.0%)
Round window	82.0 (90.1%)	69.0 (86.2%)	8.0 (100.0%)	1.0 (50.0%)
Electrode insertion status				
Complete insertion	77.0 (84.6%)	69.0 (86.3%)	7.0 (87.5%)	1.0 (50.0%)
Complete insertion with resistance	2.0 (2.2%)	5.0 (6.3%)	0.0 (0.0%)	0.0 (0.0%)
Partial insertion	12.0 (13.2%)	6.0 (7.5%)	1.0 (12.5%)	1.0 (50.0%)
Duration (months)	Duration between 1st CI and 1st revision	Duration between 1st and 2nd revision	Duration between 2nd and 3rd revision	
Mean (SD)	41.0 (38.2)	22.2 (16.8)	18.9 (7.6)	
Min, Max	2.0, 306.0	0.3, 42.5	13.5, 24.2	
No. of missing	2	0	0	

CI, cochlear implantation; SD, standard deviation.

a Detailed in Supplementary Tables S1 and S2.

The mean interval between the primary implantation and the first revision surgery was 41.0 ± 38.2 months. To study the role of intersurgical interval, subgroup analyses was performed using a 3-year cut-off interval between surgeries, as this cutoff yielded two similarly sized groups.

Comorbidities were present in 31.9% of patients. These included auditory neuropathy spectrum disorder (*n* = 3), inner ear malformations (*n* = 2), and syndromic hearing loss (*n* = 5), with the remaining comorbidities detailed in Supplementary Table S1. Complications were uncommon and are summarized in Supplementary Table S2. Surgical site infection occurred in five patients following the primary implantation and in two patients after the first revision surgery. Cholesteatoma developed in two patients after the initial implantation, and in three patients following the first revision procedure.

The round window approach was used in the majority of procedures (90.1%, *n* = 82). Complete electrode insertion without resistance was achieved in 84.6% of cases (*n* = 77). The distribution of implanted electrode arrays is presented in Table 1, while changes in electrode array type between the primary implantation and revision surgeries are summarized in Supplementary Table S3. Approximately half of the reimplantation procedures involved a change in electrode array.

### Audiological and speech outcomes across revision surgeries

Table 2 summarizes the longitudinal changes in audiological and speech outcomes following the primary CI and subsequent revision surgeries. All patients presented with profound sensorineural hearing loss in the implanted ear before the initial CI. Outcomes assessments were measured again about 18 months postoperatively.

**Table 2:** Changes in outcome measures following cochlear reimplantation surgeries.

Outcome measure	Surgery	Raw mean	EM mean	Lower limit	Upper limit	Pairwise comparisons	Estimate difference	*P* value
PTA (dB HL)	Pre CI (*N* = 91)	89.7	89.7	87.8	91.7	(First CI)—(Pre CI)	−55.6	<0.001
	First CI (*N* = 91)	34.0	34.1	31.9	36.3	(First Rev.)—(First CI)	−1.8	0.767
	First revision (*N* = 80)	32.2	32.3	30.1	34.5	(Second Rev.)—(First CI)	−1.9	0.993
	Second revision (*N* = 8)	32.6	32.2	24.0	40.5	(Second Rev.)—(First Rev.)	−0.09	1.000
	Third revision (*N* = 2)	29.0	29.8	16.8	42.7	(Third Rev.)—(First CI)	−4.3	0.967
SRT (dB HL)	Pre CI (*N* = 91)	84.4	84.0	76.4	91.7	(First CI)—(Pre CI)	−51.9	<0.001
	First CI (*N* = 91)	32.2	32.1	29.0	35.2	(First Rev.)—(First CI)	−0.2	1.000
	First revision (*N* = 80)	31.8	31.9	29.3	34.5	(Second Rev.)—(First CI)	4.7	0.890
	Second revision (*N* = 8)	37.4	36.8	27.1	46.5	(Second Rev.)—(First Rev.)	4.9	0.869
	Third revision (*N* = 2)	35.0	35.1	19.9	50.3	(Third Rev.)—(First CI)	3.0	0.995
SDS (%)	Pre CI (*N* = 91)	42.0	43.6	9.6	77.7	(First CI)—(Pre CI)	12.1	0.952
	First CI (*N* = 91)	55.8	55.7	44.8	66.7	(First Rev.)—(First CI)	8.5	0.622
	First revision (*N* = 80)	64.6	64.3	57.6	70.9	(Second Rev.)—(First CI)	−20.3	0.673
	Second revision (*N* = 8)	33.3	35.4	7.0	63.8	(Second Rev.)—(First Rev.)	−28.8	0.287
	Third revision (*N* = 2)	48.0	49.0	14.6	83.5	(Third Rev.)—(First CI)	−6.7	0.996
CAP	Pre CI (*N* = 91)	0.4	0.4	−0.2	0.9	(First CI)—(Pre CI)	5.3	<0.001
	First CI (*N* = 91)	5.6	5.6	5.1	6.2	(First Rev.)—(First CI)	0.9	0.037
	First revision (*N* = 80)	6.5	6.6	6.0	7.1	(Second Rev.)—(First CI)	−1.3	0.730
	Second revision (*N* = 8)	3.3	4.4	2.4	6.3	(Second Rev.)—(First Rev.)	−2.2	0.200
	Third revision (*N* = 2)	3.0	4.0	1.2	6.8	(Third Rev.)—(First CI)	−1.6	0.777
SIR	Pre CI (*N* = 91)	1.3	1.3	1.0	1.6	(First CI)—(Pre CI)	1.7	<0.001
	First CI (*N* = 91)	3.0	3.0	2.7	3.4	(First Rev.)—(First CI)	0.9	<0.001
	First revision (*N* = 80)	3.9	3.9	3.6	4.2	(Second Rev.)—(First CI)	0.7	0.754
	Second revision (*N* = 8)	3.5	3.7	2.6	4.8	(Second Rev.)—(First Rev.)	−0.2	0.997
	Third revision (*N* = 2)	2.0	2.4	0.8	3.9	(Third Rev.)—(First CI)	−0.7	0.906

CI, cochlear implantation; EM mean, estimated marginal mean; PTA, pure tone average; SRT, speech reception threshold; SDS, speech discrimination score; CAP, categories of auditory performance; SIR, speech intelligibility rating.

Longitudinal analysis of PTA demonstrated a significant improvement of 55.6 dB following the primary CI, with EMM decreasing from 89.7 dB HL preoperatively to 34.1 dB HL postoperatively (*p* < 0.001). PTA improvements were maintained following subsequent revision surgeries, with EMM of 32.3, 32.2, and 29.8 dB HL after the first, second, and third reimplantations, respectively. These differences were not statistically significant compared with the outcomes achieved after the primary implantation, indicating that postoperative aided audiological performance remained stable following repeated reimplantation (Table 2, Supplementary Figure S4A).

SRT similarly improved after the primary implantation, decreasing by 51.9 dB from a preoperative estimated marginal mean of 84.0 to 32.1 dB HL postoperatively (*p* < 0.001). No significant differences in SRT were observed between the initial implantation and subsequent reimplantations, suggesting sustained speech detection performance following reimplantations (Table 2, Supplementary Figure S4B).

SDS demonstrated a modest, non-significant improvement of 12.1% following the primary implantation compared with the preoperative assessment, followed by an additional non-significant increase of 8.5% after the first revision surgery. Interpretation of SDS results should be made cautiously because this assessment was not feasible in many young pediatric patients, resulting in substantial missing data (Table 2, Supplementary Figure S4C).

CAP scores increased significantly after the primary implantation, with a mean improvement of 5.3 points compared with the preoperative evaluation (*p* < 0.001). In addition, CAP scores showed a further significant improvement of 0.9 points following the first revision surgery compared with the initial implantation (*p* = 0.037), indicating continued gains in functional auditory performance after reimplantation (Table 2, Supplementary Figure S4D).

Similarly, SIR improved significantly after the primary implantation, increasing by 1.7 points compared with baseline (*p* < 0.001). A further significant improvement of 0.9 points was observed following the first revision surgery relative to the initial implantation (*p* < 0.001), demonstrating continued enhancement of speech intelligibility after reimplantation (Table 2, Supplementary Figure S4E).

### Factors affecting audiological and speech outcomes after reimplantation

Specific confounders were studied to assess their impact on postoperative outcomes, namely: device upgrade, change in electrode array type, depth of insertion, and duration between revisions.

Failure in the present cohort occurred across all three major manufacturers represented in our practice, comprising 70 MED-EL devices (Synchrony, Concerto, Sonata), 11 Cochlear devices (CI24R, CI512, CI422), and 10 Advanced Bionics devices (HiRes Ultra). The predominance of MED-EL devices among failed implants is likely attributable to the higher number of MED-EL implantations performed at our center during the study period, rather than an actual increased manufacturer-specific risk of device failure.

The role of advancement in technology was taken into consideration as a confounder to outcomes’ improvement post revision. We attempted to study internal device upgrades, however this was limited by incomplete data For Cochlear and Advanced Bionics implanted cases, which hinders tracking their upgrading status over revision surgeries.

For MED-EL implanted cases, Figure 6 illustrates that among those who undergone revision surgery (60 implants), five cases upgraded their old implants during reimplantation (4 cases from Concerto to Synchrony, and 1 case from Sonata to Concerto). Another 35 cases maintained the same old implant (Concerto), while the remaining 20 cases maintained the recent implant model (Synchrony) upon revision.

### Factors affecting PTA after reimplantation

Changing the electrode array during the first revision surgery was not associated with a significant difference in postoperative PTA compared with reimplantation using the same electrode type. Similarly, patients who underwent an electrode array change during the second revision surgery (*n* = 4) demonstrated higher postoperative PTA thresholds than those reimplanted with the same electrode type (*n* = 4); however, this difference did not reach statistical significance (Figure 1A).

**Figure 1:**
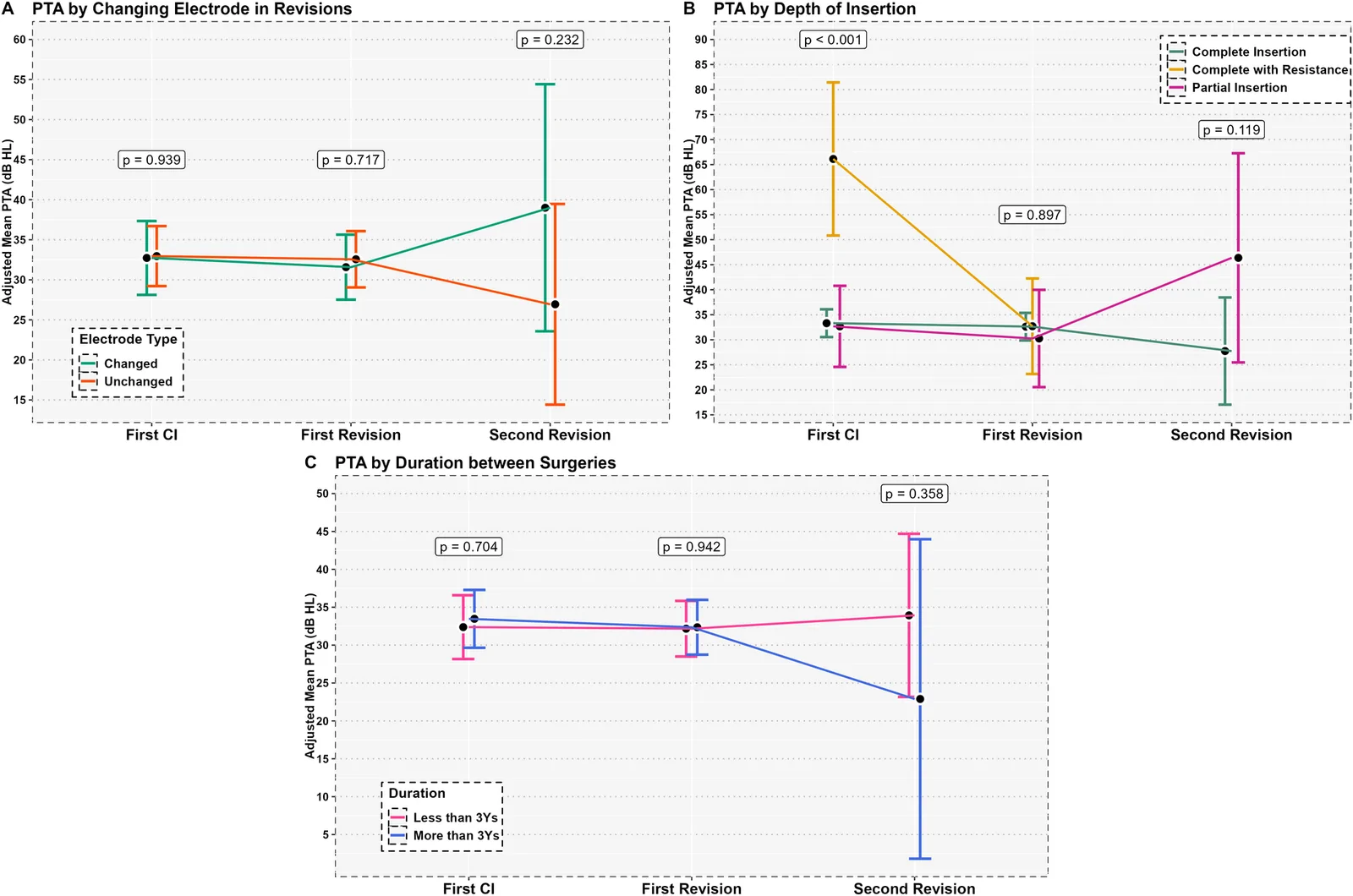
Factors affecting changes in pure tone average (PTA) across multiple cochlear re-implantation surgeries: **(A)** electrode type replacement, **(B)** electrode insertion depth with or without detectable resistance, and **(C)** interval between surgeries (years); points represent the estimated marginal mean, and error bars represent the 95% confidence interval.

Regarding insertion characteristics, cases with complete electrode insertion accompanied by intraoperative resistance during the primary implantation demonstrated higher postoperative PTA thresholds than those with complete insertion without resistance or partial insertion. However, this finding should be interpreted cautiously because only two patients exhibited insertion resistance during the primary implantation. Following the second revision surgery, partial electrode insertion was associated with higher PTA thresholds than complete insertion, although this difference was not statistically significant (Figure 1B).

The interval between implantation procedures (>3 years vs. ≤3 years) was evaluated as a potential modifier of postoperative PTA outcomes and was not associated with any statistically significant differences between the two groups (Figure 1C).

### Factors affecting SRT after reimplantation

Consistent with the PTA findings, changing the electrode array during the first revision surgery had no significant effect on postoperative SRT. In contrast, patients reimplanted with the same electrode type during the second revision demonstrated significantly better SRT thresholds than those who underwent an electrode array change (*p* = 0.022) (Figure 2A).

**Figure 2:**
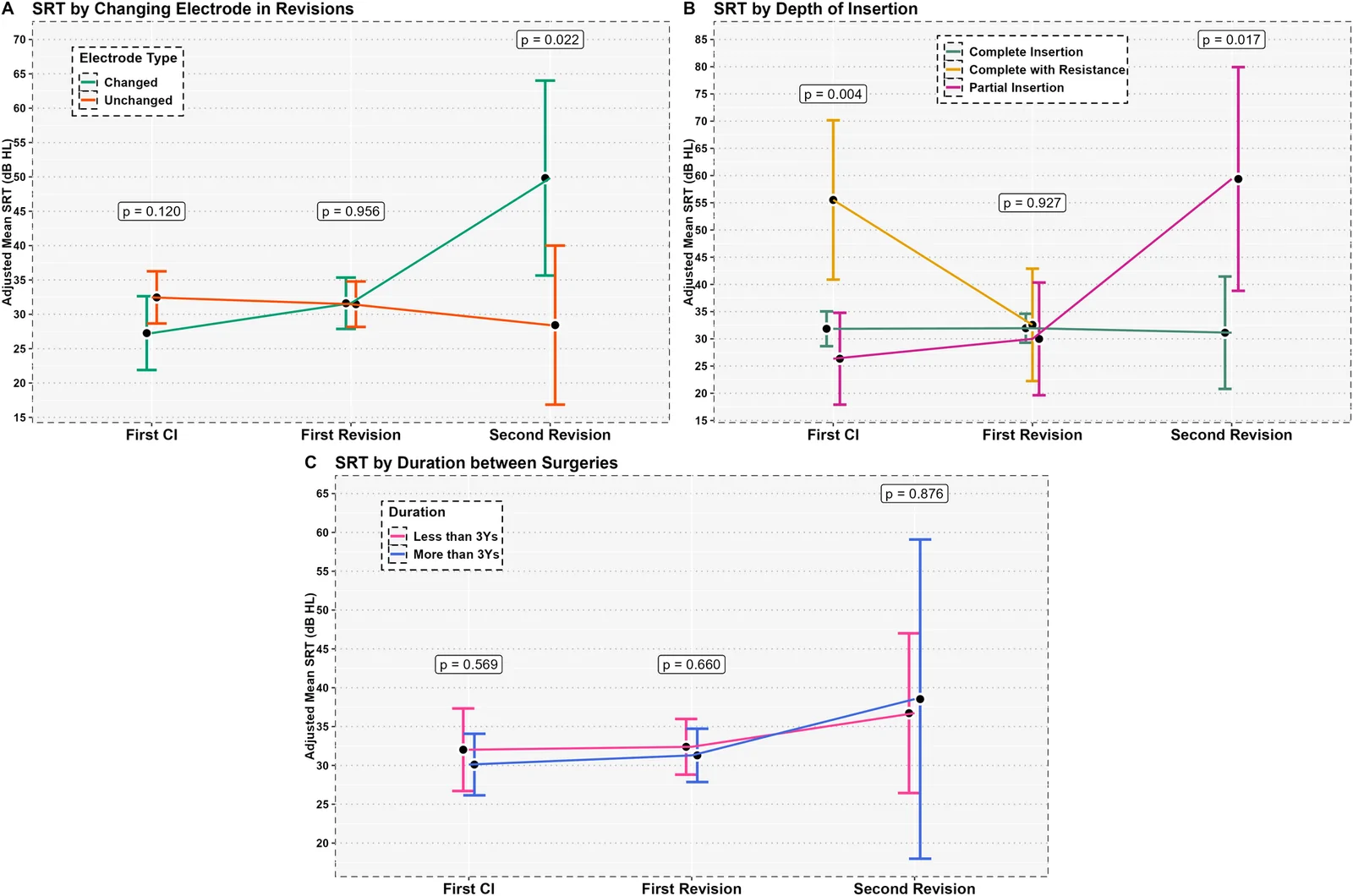
Factors affecting changes in speech reception threshold (SRT) across multiple cochlear implantation surgeries: **(A)** electrode type replacement, **(B)** electrode insertion depth with or without detectable resistance, and **(C)** interval between surgeries (years); points represent the estimated marginal mean, and error bars represent the 95% confidence interval.

Complete electrode insertion in the presence of intraoperative resistance during the primary implantation was associated with poorer postoperative SRT; however, this observation was based on only two patients, hence it should be interpreted with caution, as larger numbers is needed to support that. In addition, partial electrode insertion during the second revision surgery resulted in higher SRT thresholds than complete insertion (*p* = 0.017) (Figure 2B). The interval between surgeries had no significant effect on postoperative SRT (Figure 2C).

### Factors affecting SDS after reimplantation

Patients reimplanted with the same electrode type demonstrated higher postoperative SDS than those who underwent an electrode array change during both the first and second revision surgeries. This difference reached statistical significance following the first revision (*p* = 0.035) but not after the second revision (Figure 3A). Neither electrode insertion characteristics nor the interval between surgeries significantly influenced SDS outcomes (Figures 3B,C).

**Figure 3:**
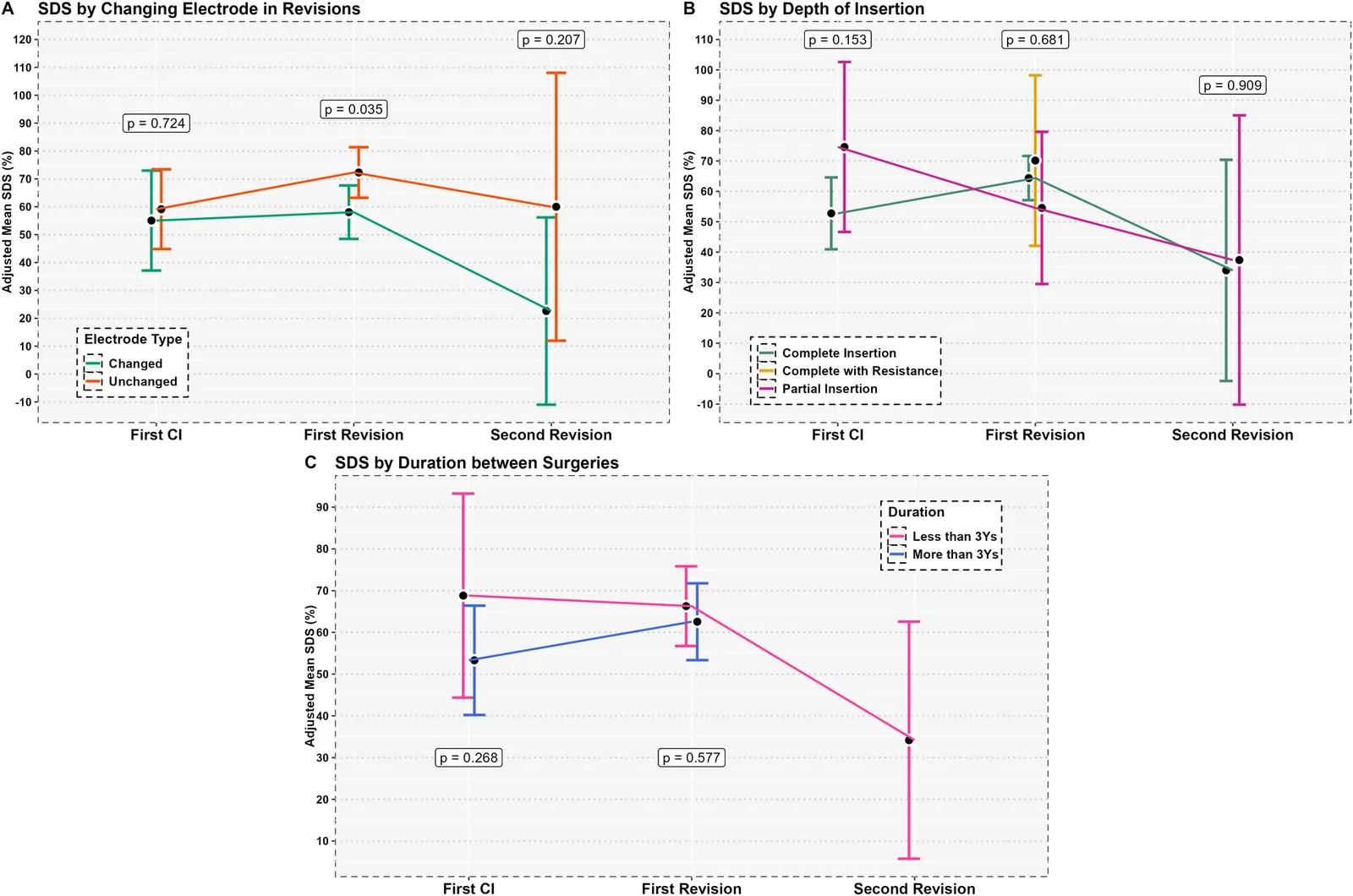
Factors affecting changes in speech discrimination scores (SDS) across multiple cochlear implantation surgeries: **(A)** electrode type replacement, **(B)** electrode insertion depth with or without detectable resistance, and **(C)** interval between surgeries (years); points represent the estimated marginal mean, and error bars represent the 95% confidence interval.

Unlike those implanted with the same implant model, the stratified analysis indicated that cases which underwent an upgrade in the internal device (*n* = 5) showed a significant improvement only in SDS (*p* = 0.021) (Figure 6).

### Factors affecting CAP after reimplantation

Electrode array replacement was associated with a non-significant increase in CAP scores after the first revision and a non-significant decrease after the second revision compared with reimplantation using the same electrode type (Figure 4A). Likewise, CAP scores were not significantly influenced by electrode insertion characteristics or the interval between surgeries (Figures 4B,C).

**Figure 4:**
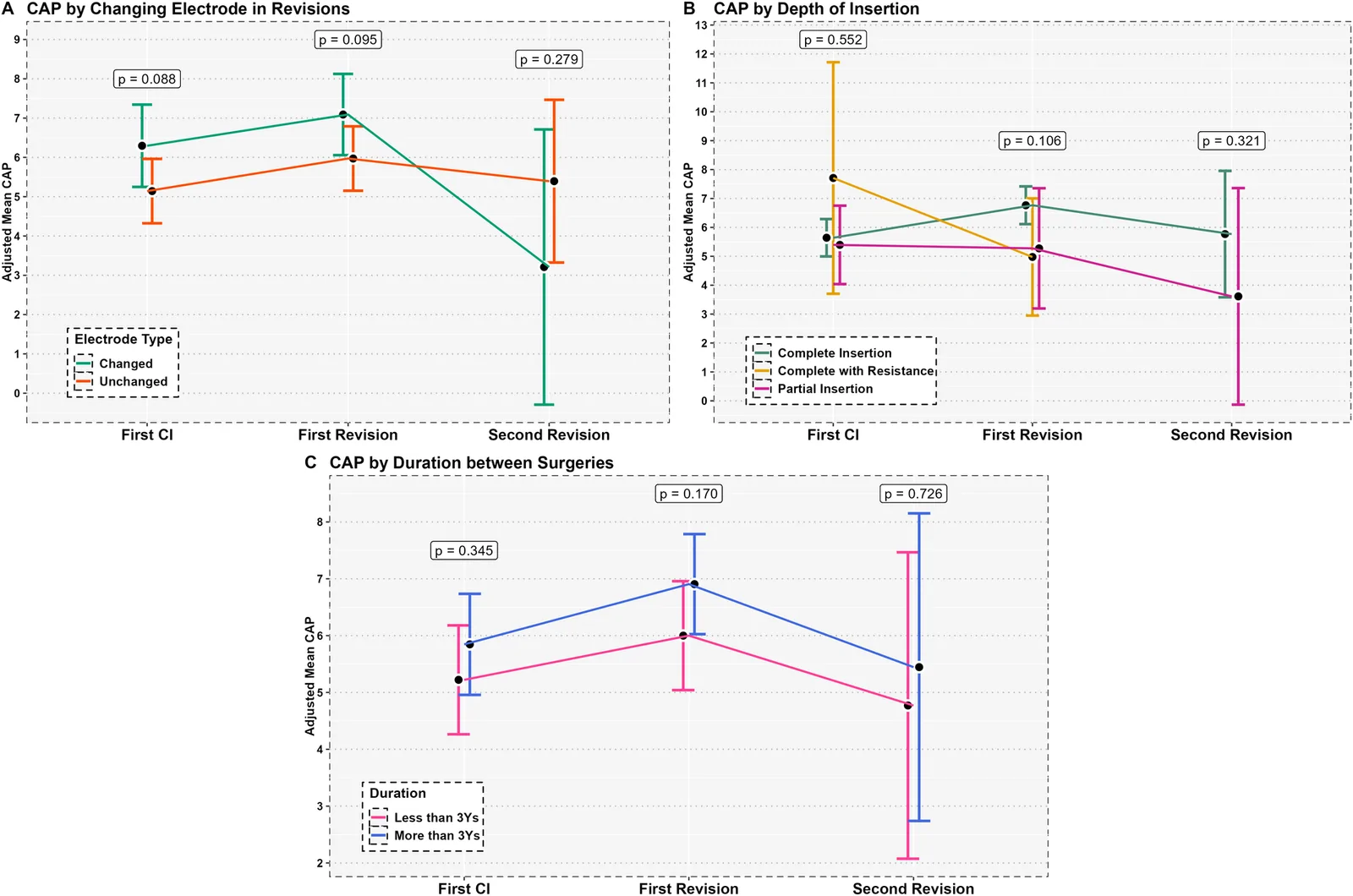
Factors affecting changes in categories of auditory performance (CAP) scores across multiple cochlear implantation surgeries: **(A)** electrode type replacement, **(B)** electrode insertion depth with or without detectable resistance, and **(C)** interval between surgeries (years); points represent the estimated marginal mean, and error bars represent the 95% confidence interval.

### Factors affecting SIR after reimplantation

Changing the electrode array and complete electrode insertion during the second revision surgery, was associated with significantly lower postoperative SIR scores, whereas no significant association was observed after the first revision (Figures 5A,B).

**Figure 5:**
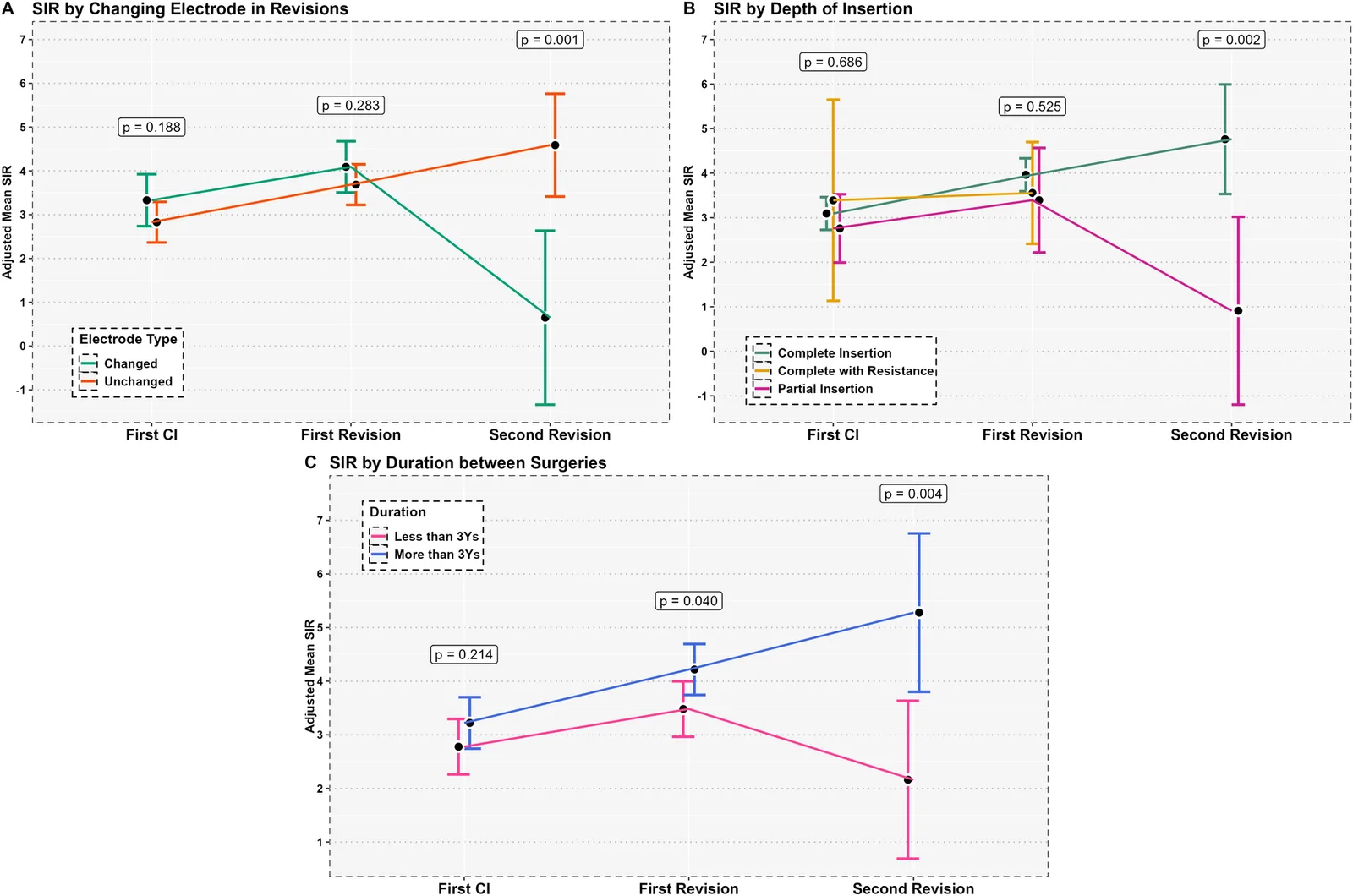
Factors affecting changes in speech intelligibility rating (SIR) across multiple cochlear implantation surgeries: **(A)** electrode type replacement, **(B)** electrode insertion depth with or without detectable resistance, and **(C)** interval between surgeries (years); points represent the estimated marginal mean, and error bars represent the 95% confidence interval.

Patients with an interval exceeding 3 years between consecutive implantation procedures achieved significantly higher postoperative SIR scores following both the first and second revision surgeries than those with intervals of 3 years or less (*p* = 0.040 and *p* = 0.004, respectively) (Figure 5C). SIR score was also sensitive enough to show a statistically significant improvement after revision surgery in all subgroups regardless of their internal device upgrade status (Figure 6).

**Figure 6:**
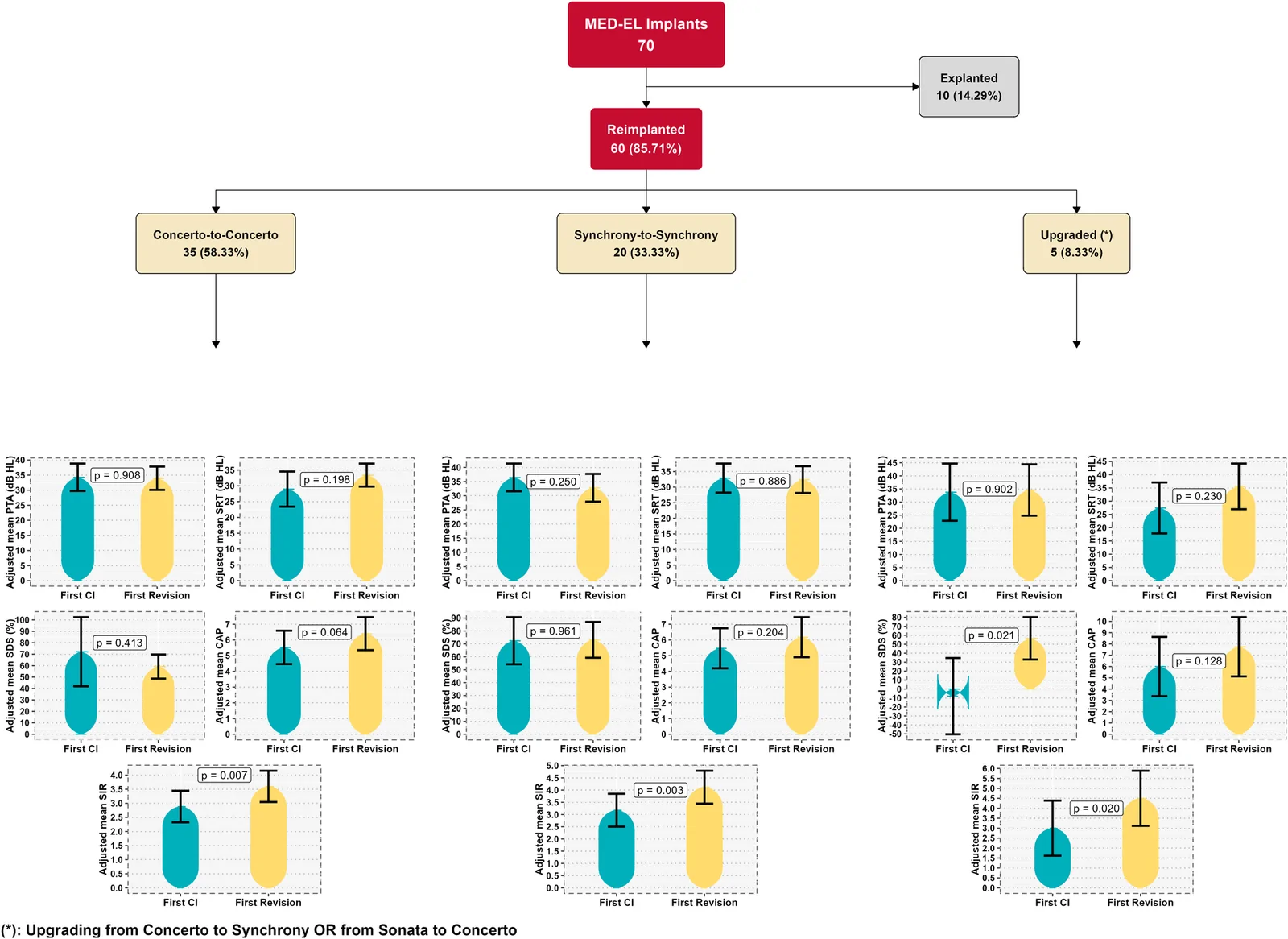
Role of implant device upgrade in changing post-CI outcomes (PTA, SRT, SDS, CAP, and SIR, respectively): bars represent the estimated marginal means, and error bars represent the 95% confidence intervals.

## Discussion

Failure of CI can erode previously achieved auditory benefit and impose considerable psychological and logistical burden on patients, their families, and the treating institution. Although revision surgery remains the definitive management for device failure, the question of how many revisions should reasonably be offered to a given patient remains unresolved in the literature. The present series extends an earlier institutional report of 922 CI cases with a revision rate of 4% (3). A cumulative of 3,777 implantations were performed at our centre over 31 years, of which 91 (2.41%) required revision. This failure rate is consistent with contemporary multi-institutional experience, in which reported rates of revision cochlear implantation range from approximately 2.6%–11.7%, while rates attributable specifically to device failure range from 1.9% to 8.1% (11, 12).

Hard device failure accounted for the majority of revisions (68.8%), in keeping with previously published series (2). Meanwhile, Woo Nam et al. examined revisions after 2012 and noted soft/functional failures to be the most common indication for reimplantation, and linked the decrease in hard device failures to enhanced device engineering and electrode configurations in the modern era of CI (13). Although device failure was historically presumed to be more frequent in children owing to greater exposure to external head trauma, the literature has not substantiated a meaningful difference in implant survival between paediatric and adult recipients (2).

Long-term surveillance following CI is essential, as re-implantation has been reported to occur anywhere from 2 to 4 years, and occasionally more than a decade after the index procedure (6). It should be emphasised that the date of re-implantation is an imperfect proxy for the true date of device failure, since the onset of failure—particularly in children and in cases of soft failure—is often difficult to establish with precision. Diagnostic delay may be compounded by lower parental health literacy and socioeconomic disadvantage, which can postpone recognition of device malfunction and subsequent presentation for evaluation. In the present cohort, the interval between primary implantation and first revision averaged 41 months, whereas the interval between subsequent revisions was shorter (18.9–22.2 months). This may reflect heightened parental vigilance following a first re-implantation experience, which could plausibly reduce diagnostic and referral delay should further device issues arise.

Revision surgery carries its own procedural challenges and complication risks, which must be weighed against the benefits of restoring auditory function. The implant bed and mastoid cavity frequently undergo neo-osteogenesis, particularly in paediatric patients, necessitating additional operative time and modified surgical technique (14). Cochlear ossification has similarly been described in the literature as a barrier to complete electrode re-insertion at revision, with attendant effects on perceptual outcome (7, 8, 15). In the present cohort, only four cases developed mild labyrinthine ossification after the initial implantation.

Of note, complications rate has varied from 19.8% (*n* = 18 of 91) at the first CI to 15% (*n* = 12 of 80) at the first revision. The literature also showed that revision cochlear implantation, while not without risk, is a comparatively safe undertaking when performed at high-volume centres (16).

### Factors affecting revision outcomes

The functional outcome of re-implantation has been debated in the literature, with several reports suggesting that rehabilitation after revision does not consistently match the effectiveness achieved after the initial implantation (8, 17). Reis et al., in contrast, demonstrated that SDS in adults improved after re-implantation to a level comparable with the best SDS recorded prior to revision (15). Poor performance following revision has been associated with medical aetiologies such as inner ear malformation, cochlear ossification, and central nervous system disease (7), whereas hard device failure has been associated with more favourable outcomes after re-implantation (15).

#### Inter-surgical duration

In the present cohort, re-implantation preserved the audiological and speech gains established after the primary implantation across all measured parameters—PTA, SRT, SDS, CAP, and SIR. Both CAP and SIR demonstrated further significant improvement after the first revision (*p* = 0.037 and *p* < 0.001, respectively), consistent with prior reports (6, 7). This additional gain is most plausibly attributable to maturation of the central auditory pathway, cumulative rehabilitative exposure, and ongoing cognitive development within a predominantly paediatric cohort, rather than an inherent technical superiority of the replacement device. This interpretation is reinforced by the observation that patients with an inter-surgical interval exceeding 3 years demonstrated significantly higher SIR scores after both the first and second revisions (*p* = 0.040 and *p* = 0.004, respectively) compared with those revised within 3 years—bearing in mind that roughly half of our patients had a successfully implanted contralateral ear (46.15%) or single-sided deafness (5.49%). These findings are further limited by incomplete audiological data, particularly for SDS, given that 96.6% of the cohort was paediatric.

#### Changing the electrode type

Choice of device manufacturer in this cohort reflected a shared decision between surgeon and patient, whereas electrode selection was guided by cochlear anatomy, cochlear duct length, gusher risk, and the presence of malformation or ossification. In 37 cases, the electrode array was changed to a different type at the time of revision. The dominant pattern observed was a shift from perimodiolar to lateral-wall electrodes, and from longer, older-generation arrays to shorter contemporary designs; while only two cases involved a change of implant manufacturer (Supplementary Table S3).

Theoretically, the change to a shorter electrode can limit optimal neural stimulation (8), though that might have been unavoidable facing cochlear fibrosis in revision. Additionally, revisions with perimodilar electrodes rather than lateral-wall electrodes was associated with significant gains in speech perception, likely due to superior modiolar proximity and re-insertion overcoming cochlear fibrosis (13).

Electrode substitution did not, overall, confer a demonstrable outcome advantage. On the contrary, three parameters have shown statistically significant difference between the groups, favouring the use of similar electrode type. SRT thresholds improved among patients who did not undergo a change in electrode type during the second revision surgery (*p* = 0.022) (Figure 2A). Patients re-implanted with the same type of electrode during the first revision surgery demonstrated statistically significant SDS improvement, though it was not clinically significant compared with those who underwent electrode type replacement (70% and 60%, respectively) (Figure 3A). SIR scores were likewise higher in the unchanged-electrode group after the second revision (*p* = 0.001), albeit with a substantial proportion of missing SIR data (*n* = 2) that warrants cautious interpretation (Figure 5A). Further analysis and interpretation could not be performed due to the wide range of electrode types used in this study.

#### Electrode insertion depth and resistance

Although higher PTA and SRT thresholds were observed after the initial implantation among the small number of cases with complete insertion accompanied by intraoperative resistance—relative to those with unimpeded complete insertion or partial insertion—this finding is of limited reliability, as only two cases met this specific combination of criteria at primary implantation. No comparable difference emerged after the first revision. However, partial insertion was associated with significantly worse SRT (*p* = 0.017) and SIR (*p* = 0.002) outcomes after the second revision. Though the current paper does not radiologically assess electrode position and insertion angle, radiological observations of Roßberg et al. showed that re-implantation is associated with a reduced insertion angle, diminished cochlear coverage, and shorter effective intracochlear electrode length (8). Consequently, the efficiency of complex acoustic coding strategies is compromised, reinforcing that achieving maximum insertion depth is helpful in speech clarity and structural sound plasticity of CI (8, 18).

#### Internal device upgrades

Our results demonstrate that patients who received upgraded internal hardware upon revision (e.g., transitioning from Concerto to Synchrony) achieved statistically significant gains in SDS and SIR post-reimplantation. This functional improvement is likely attributable to advancements in recent implant generations, including optimized electrode designs, superior signal processing capabilities, and refined mechanical properties that facilitate atraumatic insertion and enhanced neural interface fidelity.

Interestingly, while SDS gains were contingent upon receiving an upgraded internal model, SIR scores showed statistically significant improvements across all revision cases regardless of upgrade status. This suggests that SIR—a measure of functional daily speech clarity—may benefit from the combined effect of secondary surgical optimization and continued post-revision auditory rehabilitation, whereas acoustic resolution metrics like SDS are more constrained by internal hardware performance.

Despite the inherently small number of those who underwent revision surgeries, we cannot ignore the detected associations, especially upon performing the MMRM analysis and assuming a compound symmetry covariance structure that complies with the small sample. However, the related findings should be treated as exploratory investigations.

### Special considerations

#### Explantation

Eleven cases underwent explantation after the primary implantation without subsequent re-implantation, at a mean age of 5.3 ± 3.5 years; only four of these patients were female. Among these, five had been implanted with a Flex28 electrode, two with Form24, and one each with Flex24, Medium, Standard, and SlimJ arrays. Six of the eleven explanted patients had bilateral implants, one of whom experienced bilateral device failure, and one case with SSD. Explantation was most frequently prompted by medical causes—infection or cholesteatoma—(*n* = 9), with soft failure and hard failure each accounting for a single case each. Prior to explantation, these patients had derived measurable audiological benefit from implantation, with mean PTA improving from 90 dB HL preoperatively to 42.6 dB HL postoperatively, and modest gains in CAP and SIR scores, from preoperative means of 1.5 and 1.7 points to postoperative means of 5.8 and 3.0 points, respectively.

#### Single-sided deafness (SSD)

Device failure occurred in five paediatric patients with SSD (three right-sided, two left-sided), with a mean age at implantation of 6.35 ± 5.36 years (three females, two males). Four of the five underwent a first revision 19.7 months from primary implantation. The fifth patient, who reported no perceptible benefit from the original device, declined re-implantation. Only one of the four revised cases required a second revision, necessitated by a fractured electrode array. Audiological assessment in this subgroup demonstrated improvement in mean PTA from 90 dB HL preoperatively to 20.0 dB HL after the first implantation and 22.5 dB HL after the first revision. Mean SRT was 25 dB HL, and mean SDS reached 66% following re-implantation.

### Limitations

This study is limited primarily by its retrospective design and the inherent attrition associated with long-term follow-up. As the study spans more than three decades (1993-2024), technology advances may have influenced both device failure patterns and functional outcomes. In addition, findings related to the second and third revision surgeries should be interpreted with caution, as the limited sample size (*n* = 8 and *n* = 2 respectively) and increasing proportion of missing data reduce the statistical power and limit the generalisability of these results. Furthermore, the predominantly pediatric composition of the cohort contributed to incomplete outcome data, as several audiological and speech perception measures are age-dependent and cannot be reliably administered to very young children. Lastly, comparison of device failure ratio per company was not feasible, as some patients' data were lost, specifically for Cochlear LTD.

Future prospective studies utilizing multi-center registries with comprehensive documentation are needed to confirm whether these functional advantages can be generalized.

In conclusion, CI failure remained uncommon, with an overall prevalence of 2.41% over more than three decades. Reimplantation is a salvage procedure that successfully restores auditory function after device failure, allowing for continuous language development. Electrode selection and revision strategy should be individualized according to patient and surgical factors.

## Data Availability

The raw data supporting the conclusions of this article will be made available by the authors, without undue reservation.
