# Supplementary material for: Three decades of cochlear implant failure and multiple reimplantation: a retrospective cohort study
Source: Front Surg. 2026 Oct 20;13:1941723. doi: 10.3389/fsurg.2026.1941723 (PMC13727629; doi:10.3389/fsurg.2026.1941723)
Supplement: Supplementary file 1 [file Datasheet1.docx]

## Supplementary material

**Supplementary table (S1):**

**Distribution of Comorbidity History Among Cochlear Implant Failure Cases:**

| **Comorbidity** | **Overall (N =91)** |
| --- | --- |
| Attention-Deficit/Hyperactivity Disorder (ADHD) | 1.0 (1.1%) |
| Autism | 1.0 (1.1%) |
| Albinism | 1.0 (1.1%) |
| Bronchial Asthma | 3.0 (3.3%) |
| Gastroesophageal reflux disease (GERD) | 1.0 (1.1%) |
| Hyperprolactinemia | 1.0 (1.1%) |
| Brain atrophy | 1.0 (1.1%) |
| Cerebral palsy | 2.0 (2.2%) |
| Hypertension | 1.0 (1.1%) |
| Brucellosis | 1.0 (1.1%) |
| Eczema | 1.0 (1.1%) |
| Glucose-6-phosphate dehydrogenase (G6PD) deficiency | 1.0 (1.1%) |
| Auditory neuropathy | 3.0 (3.3%) |
| Prolonged QT syndrome | 2.0 (2.2%) |
| Diabetes | 2.0 (2.2%) |
| Iron-Deficiency Anemia | 2.0 (2.2%) |
| Mondini dysplasia | 4.0 (4.4%) |
| Chronic Suppurative Otitis Media (CSOM) | 3.0 (3.3%) |
| Otitis Media with Effusion (OME) | 3.0 (3.3%) |
| Usher syndrome | 2.0 (2.2%) |
| Hyperinsulinemia | 1.0 (1.1%) |
| Waardenburg syndrome | 1.0 (1.1%) |
| **N.B. some of the reported cases had multiple comorbidities (n ≠ total).** | |

**Supplementary table (S2): Distribution of intraoperative and postoperative complications grouped by surgery:**

| **Complications** | **First CI  N = 18** | **First Revision  N = 12** | **Second Revision  N = 1** | **Third Revision  N = 1** |
| --- | --- | --- | --- | --- |
| Brain herniation | 1.0 (5.6%) | 0.0 (0.0%) | 0.0 (0.0%) | 0.0 (0.0%) |
| Facial nerve palsy | 1.0 (5.6%) | 0.0 (0.0%) | 0.0 (0.0%) | 0.0 (0.0%) |
| Cholesteatoma | 2.0 (11.1%) | 3.0 (25.0%) | 0.0 (0.0%) | 0.0 (0.0%) |
| Electrode migration | 2.0 (11.1%) | 0.0 (0.0%) | 1.0 (100.0%) | 0.0 (0.0%) |
| Chorda tympani injury | 1.0 (5.6%) | 1.0 (8.3%) | 0.0 (0.0%) | 0.0 (0.0%) |
| Gusher | 5.0 (27.8%) | 2.0 (16.7%) | 0.0 (0.0%) | 0.0 (0.0%) |
| Surgical Site Infection | 5.0 (27.8%) | 2.0 (16.7%) | 0.0 (0.0%) | 0.0 (0.0%) |
| Labyrinthine ossificans | 2.0 (11.1%) | 1.0 (8.3%) | 0.0 (0.0%) | 1.0 (100.0%) |
| Keloid | 0.0 (0.0%) | 1.0 (8.3%) | 0.0 (0.0%) | 0.0 (0.0%) |
| Skin necrosis | 1.0 (5.6%) | 0.0 (0.0%) | 0.0 (0.0%) | 0.0 (0.0%) |

**N.B. some of the reported cases had multiple complications (n ≠ total).**

**Supplementary table (S3): Tracking of Electrode Type Changes During Revision Surgeries:**

| **Changed electrode types (n=37)** | | | |
| --- | --- | --- | --- |
| **Patient ID** | **From** | **To** | **at which revision** |
| Patient 5 | MS | Flex24 | 1^st^ |
| Patient 9 | MS | SlimJ | 1^st^ |
| Patient 11 | Flex28 | Flex26 | 1^st^ |
| Patient 12 | Flex28 | Form24 | 2^nd^ |
| Patient 12 | Form24 | SS | 3^rd^ |
| Patient 13 | Flex28 | Form24 | 1^st^ |
| Patient 23 | Form24 | Flex28 | 1^st^ |
| Patient 28 | Medium | Form24 | 1^st^ |
| Patient 30 | Flex28 | Form24 | 1^st^ |
| Patient 37 | Standard | Flex28 | 1^st^ |
| Patient 40 | Flex28 | Form24 | 1^st^ |
| Patient 43 | Flex28 | Form24 | 2^nd^ |
| Patient 47 | Flex24 | Form24 | 1^st^ |
| Patient 48 | Standard | Flex28 | 1^st^ |
| Patient 49 | Flex24 | Form24 | 1^st^ |
| Patient 51 | Flex24 | Flex28 | 1^st^ |
| Patient 54 | Medium | Form24 | 1^st^ |
| Patient 57 | Standard | Form24 | 1^st^ |
| Patient 58 | Flex24 | Form24 | 1^st^ |
| Patient 60 | Standard | Form24 | 1^st^ |
| Patient 64 | Medium | Flex28 | 1^st^ |
| Patient 65 | Medium | Form24 | 1^st^ |
| Patient 66 | Flex28 | Form24 | 1^st^ |
| Patient 67 | Flex28 | Form24 | 1^st^ |
| Patient 69 | Medium | Form24 | 1^st^ |
| Patient 70 | Flex28 | Form24 | 1^st^ |
| Patient 71 | Flex28 | Standard | 1^st^ |
| Patient 72 | Flex28 | Standard | 1^st^ |
| Patient 74 | Flex24 | Medium | 1^st^ |
| Patient 75 | Flex28 | Standard | 1^st^ |
| Patient 77 | Form24 | Flex24 | 1^st^ |
| Patient 77 | Flex24 | Form24 | 2^nd^ |
| Patient 78 | Standard | Medium | 1^st^ |
| Patient 82 | CA | SS | 1^st^ |
| Patient 83 | CA | SS | 1^st^ |
| Patient 87 | CA | SS | 1^st^ |
| Patient 90 | CA | SS | 2^nd^ |

**Supplementary Figure (S4): The Overall Changes in Postoperative Outcome Measures Across Multiple Cochlear Re-Implantation Surgeries:**

(A) Pure Tone Average (PTA), (B) Speech Reception Threshold (SRT), (C) Speech Discrimination Scores (SDS), (D) Categories of Auditory Performance (CAP) Scores, and (E) Speech Intelligibility Rating (SIR);
the points represent the estimated marginal means, and the error bars represent the 95% confidence interval.
